# Supplementary material for: Global, Regional, and National Burden of Road Injuries from 1990 to 2019
Source: Int J Environ Res Public Health. 2022 Dec 8;19(24):16479. doi: 10.3390/ijerph192416479 (PMC9779128; doi:10.3390/ijerph192416479)
Supplement: Supplementary file 1 [file ijerph-19-16479-s001.zip › Supplementary figures.pdf]

Supplementary

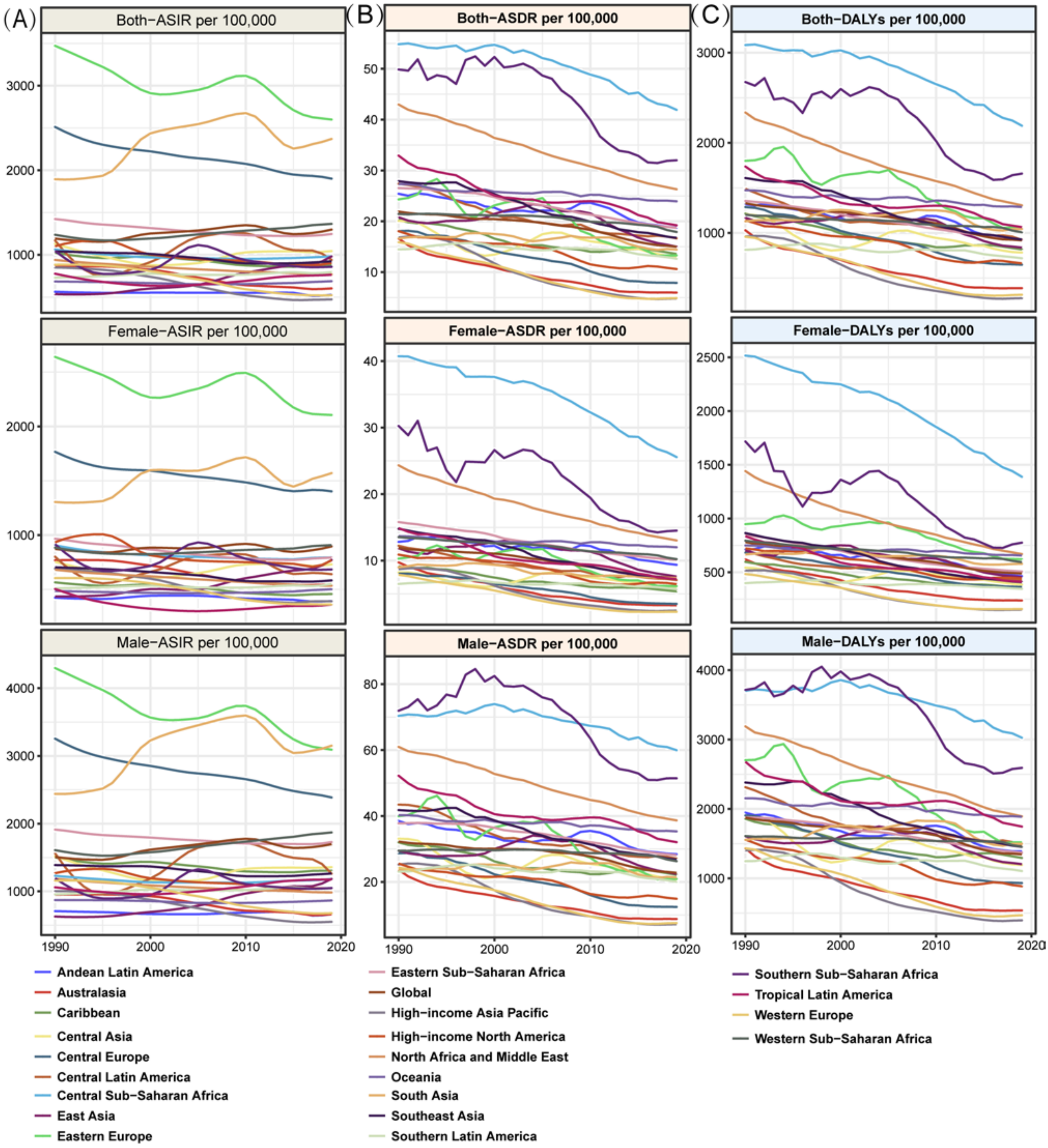

Figure S1. RI-caused burden in GBD regions from 1990 to 2019 in all populations, in women, and in men calculated by incidence (**A**), deaths (**B**), and DALYs (**C**).

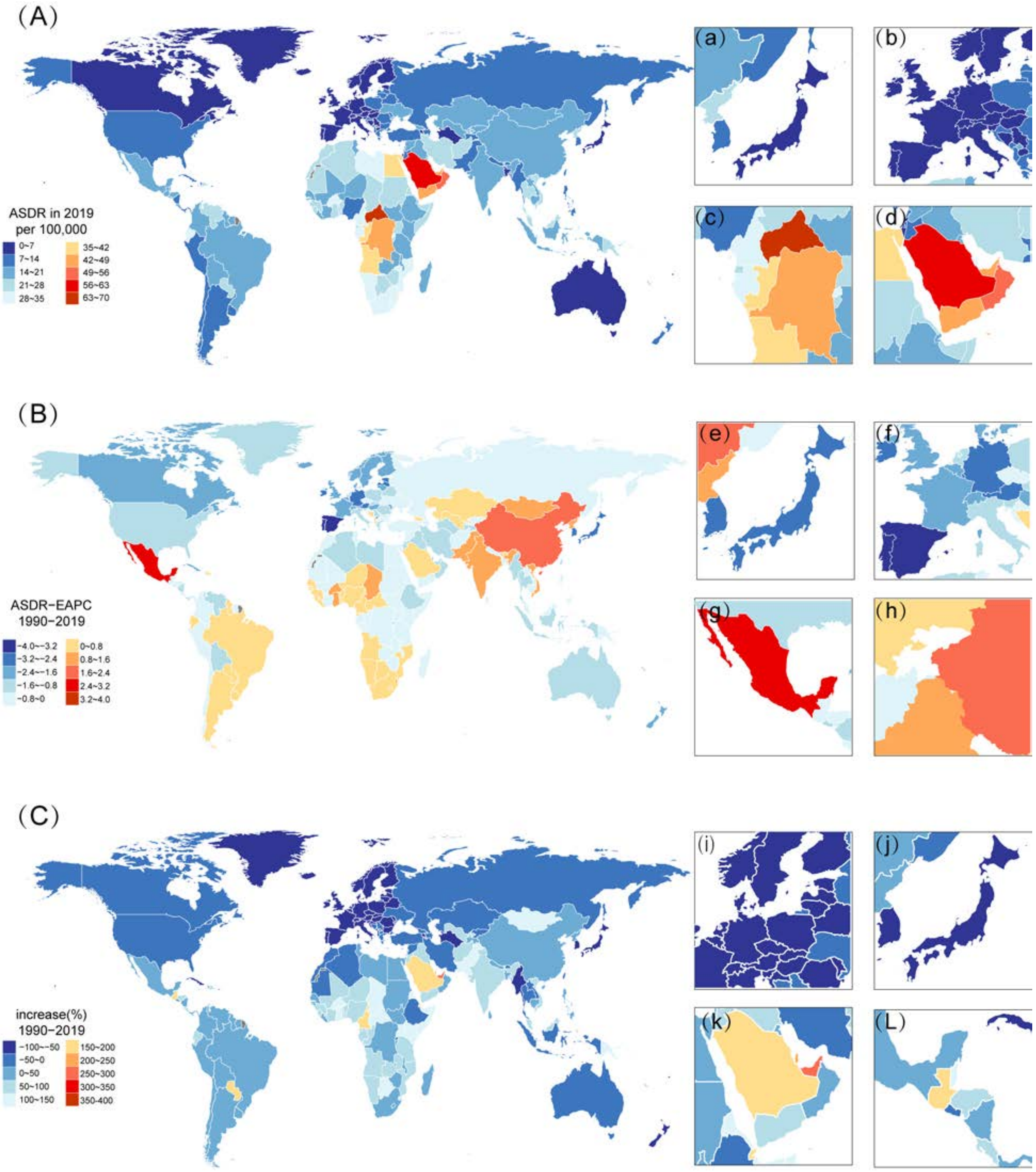

Figure S2. Death rate and frequency from 1990 to 2019 among 204 countries and territories. (**A**) Age-standardized deaths rate (ASDR) in 2019. (**B**) EAPC in ASDR. (**C**) Increase in the absolute number of deaths (%). Some high-income Asian Pacific (**a**) and Western European countries (**b**) with lower ASDR; central sub-Saharan Africa (**c**) and North Africa and the Middle East (**d**) with higher ASDR. Some high-income Asian Pacific (**e**) and Western European countries (**f**) with lower ASDR-EAPC; central Latin America (**g**) and West Asia (**h**) with higher ASDR-EAPC. Western European (**i**) and some high-income Asian Pacific countries (**j**) with a lower increase in the number of deaths; North Africa and the Middle East (**k**) and central Latin America (**l**) with a higher increase in the number of deaths.

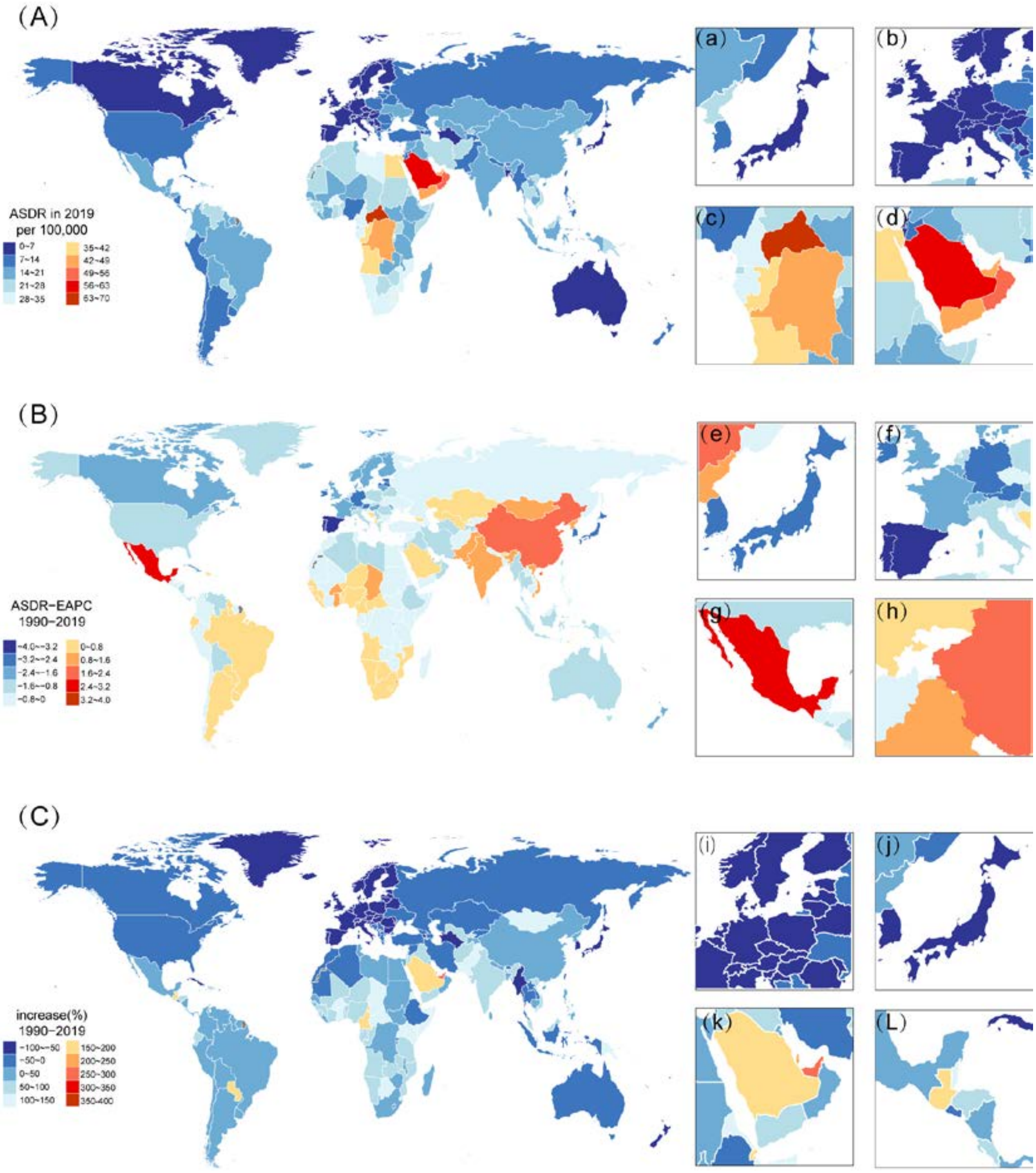

Figure S3. DALYs and associated tendencies from 1990 to 2019 among 204 countries and territories. (**A**) Age-standardized DALYs rate (AS-DALYs) in 2019. (**B**) EAPC in AS-DALYs. (**C**) Increase in the absolute number of deaths (%). Western European (**a**) and some high-income Asian Pacific countries (**b**) with lower AS-DALYs; central sub-Saharan Africa (**c**) and North Africa and the Middle East (**d**) with higher AS-DALYs. Some high-income Asian Pacific (**e**) and Western European countries (**f**) with lower ASDR-EAPC; central Latin America (**g**) and South Asia (**h**) with higher ASDR-EAPC. Western European (**i**) and some high-income Asian Pacific countries (**j**) with a lower increase in the number of deaths; North Africa and the Middle East (**k**) and central Latin America (**l**) with a higher increase in the number of deaths.
